# Rosai-Dorfman disease mimicking molluscum contagiosum in a patient with prior kappa light chain multiple myeloma

**DOI:** 10.1016/j.jdcr.2025.11.059

**Published:** 2026-03-10

**Authors:** Sharen Rivas, Nicholas Li, Daniel S. Alicea, Tyler M. Andriano, Hatice Zengin, Bijal Amin, Beth N. McLellan

**Affiliations:** Division of Dermatology, Department of Medicine, Albert Einstein College of Medicine, Montefiore Medical Center, Bronx, New York

**Keywords:** cutaneous Rosai-Dorfman disease, molluscum contagiosum, multiple myeloma, non-Langerhans cell histiocytosis, Rosai-Dorfman disease

## Case description

**Fig 1:**
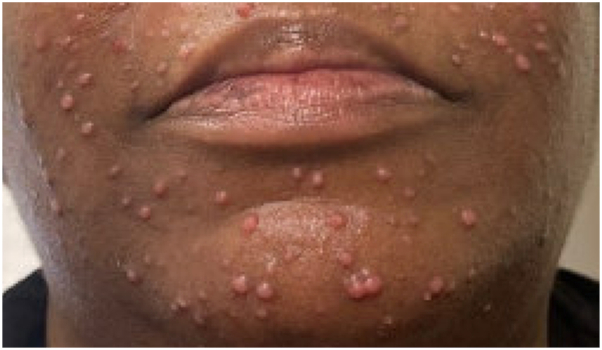
Clinical photograph of the patient’s face demonstrating numerous diffuse pink papules, several with central umbilication.

**Fig 2:**
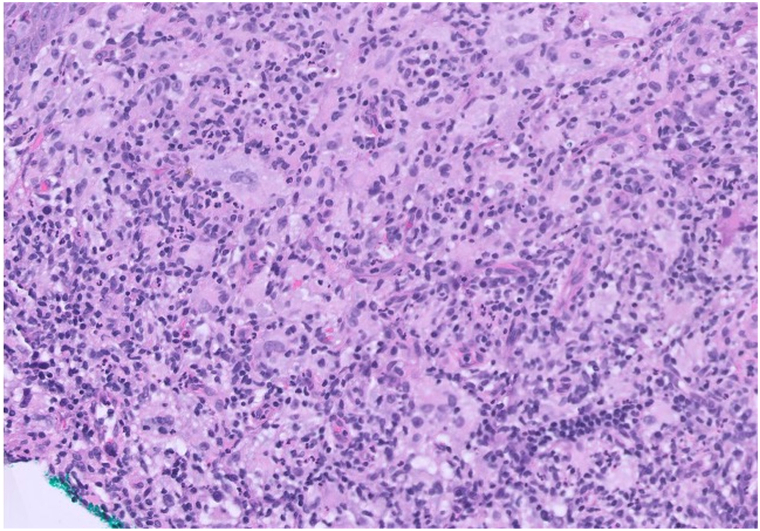
H&E stain of repeat shave biopsy from the left chin 4 months later (20× magnification), highlighting a dense histiocytic infiltrate with accompanying neutrophils, lymphocytes, and plasma cells. *H&E*, Hematoxylin and eosin.

**Fig 3:**
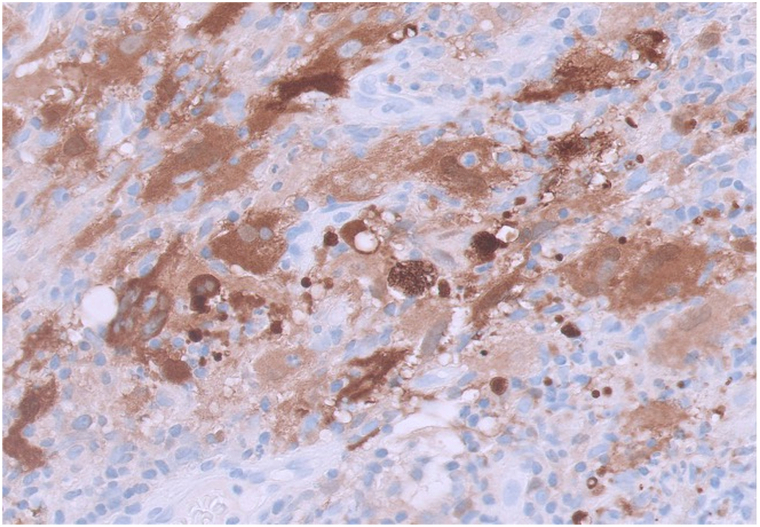
S100 immunostaining of the lesional tissue (40× magnification) is positive for histiocytes exhibiting emperipolesis, consistent with a histiocytic infiltrate.

A 53-year-old woman presented with 2 months of a nontender, nonpruritic facial rash. Her past medical history is notable for type II diabetes mellitus, hypercholesterolemia, hypertension, osteoarthritis of the right knee, and kappa light chain multiple myeloma (MM), status postautologous stem cell transplant, on active surveillance without maintenance. At rash onset, kappa light chains were 25.0 mg/L (λ 17.9 mg/L; κ/λ 1.40). At evaluation, they were 36.6 mg/L (λ 20.8 mg/L; κ/λ 1.76) and serum protein electrophoresis remained negative. Physical examination revealed diffuse pink papules on the face, some with central umbilication (Fig 1). The patient denied constitutional symptoms, and there was no cervical, axillary, or inguinal lymphadenopathy. A shave biopsy from the chin demonstrated a dense histiocytic infiltrate with accompanying neutrophils, lymphocytes, and plasma cells (Fig 2). Immunohistochemistry showed CD68+/S100+/CD1a-, and histiocytes exhibited emperipolesis (Fig 3).

**Question: Which of the following is the most likely diagnosis?**

**1.** Multicentric reticulohistiocytosis
**2.** Molluscum contagiosum
**3.** Cutaneous Rosai-Dorfman disease
**4.** Langerhans cell histiocytosis
**5.** Papular cutaneous sarcoidosis

## Answer and discussion

3. Cutaneous Rosai-Dorfman disease (CRDD) is a rare non-Langerhans cell histiocytosis that presents as red-brown or yellow papules, nodules, or plaques, sometimes with molluscum-like umbilication.^1^ Its etiology remains unknown but has been linked to aberrant immune responses to viral antigens (Epstein-Barr virus, herpes simplex virus, human herpesvirus 6, parvovirus B19, or polyomavirus).^1^, ^2^, ^3^ CRDD has been implicated in individuals with lymphoma, polyclonal hypergammaglobulinemia, and other neoplasms and autoimmune disorders.^2^ Although historically considered benign, activating KRAS/NRAS/BRAF mutations suggest a neoplastic component.^4^ These pathways are also involved in MM, which may underlie the emerging association between CRDD and MM.^4^^,^^5^

Histopathologic and immunohistochemical analyses are definitive for diagnosis. Histologically, CRDD features large histiocytes with vesicular nuclei and abundant eosinophilic cytoplasm in the dermis with accompanying emperipolesis (engulfment of inflammatory cells such as lymphocytes or plasma cells).^1^^,^^4^ Immunohistochemically, CRDD is characterized by positive CD68/S100/cyclin D1/OCT2 and negative CD1a/langerin/ALK.^1^^,^^4^ Recognition is important even when lesions regress. CRDD may cause cosmetic and functional morbidity, may indicate systemic disease, and is associated with hematologic neoplasms, including MM. CRDD generally follows a benign course with spontaneous resolution in 24% of cases and recurrence in 4%.^1^ Observation is reasonable for asymptomatic disease. When treatment is needed, however, options include cryotherapy, acitretin, intralesional corticosteroids, methotrexate, radiation, chemotherapy, or excision, with 10% to 30% response rates.^1^^,^^3^

In adults with a history of hematologic malignancy, new monomorphic, umbilicated facial papules should prompt early biopsy to exclude histiocytoses such as CRDD. Clinically, CRDD may mimic molluscum contagiosum; diagnosis hinges on histopathology and an S100+/CD68+/CD1a profile with emperipolesis, which distinguishes CRDD from Langerhans cell histiocytosis (CD1a+/langerin+), multicentric reticulohistiocytosis (CD68+/S100-), and papular cutaneous sarcoidosis (noncaseating granulomas).

Our case highlights an atypical presentation of CRDD with molluscum-like clinical mimicry in a patient with skin of color. We also emphasize biopsy triggers in adults with hematologic malignancies and the diagnostic immunophenotype (S100+/CD68+/CD1a-) of CRDD. The association of CRDD with MM remains limited.^4^^,^^5^ Given our patient’s remission status at presentation, no association can be inferred in this case.

## Conflicts of interest

None disclosed.
